# Digital Health Among Educators: A Scoping Review

**DOI:** 10.3390/healthcare14193313

**Published:** 2026-10-04

**Authors:** Weiyan Liao, Min Qiu, Dandan Wu, Xianhan Huang, Junjun Chen, Minjuan Wang, Hui Li

**Affiliations:** 1 Department of Early Childhood Education, The Education University of Hong Kong, 10, Lo Ping Road, Tai Po, Hong Kong SAR, China; 2 AI, Brain and Child Research Centre, Academy for Educational Development and Innovation, The Education University of Hong Kong, 10, Lo Ping Road, Tai Po, Hong Kong SAR, China; 3 Department of Curriculum and Instruction, The Education University of Hong Kong, 10, Lo Ping Road, Tai Po, Hong Kong SAR, China; 4 Department of Education Policy and Leadership, The Education University of Hong Kong, 10, Lo Ping Road, Tai Po, Hong Kong SAR, China; 5 Department of Mathematics and Information Technology, The Education University of Hong Kong, 10, Lo Ping Road, Tai Po, Hong Kong SAR, China

**Keywords:** digital health, ehealth, teacher education, educational technology, scoping review

## Abstract

**Background:** Digital health technologies are increasingly used to support health management, monitoring, and health-related practices. Educators face a range of physical and mental health challenges, yet research on educators’ digital health remains fragmented across educational settings, geographical contexts, and forms of digital health management. This scoping review aimed to map and synthesize the literature on educators’ digital health by examining (1) digital health literacy and related competencies; (2) experiences with digital health technologies, and (3) factors influencing educators’ capacity to navigate the digital health ecosystem. **Methods:** The scoping review was conducted in accordance with the PRISMA-ScR guidelines. A systematic search was conducted for English written empirical peer-reviewed articles related to educators’ digital health on 8 June 2026. **Results:** A total of 31 studies were included. Three themes were identified. First, educators’ digital health literacy varied across demographic groups and educational settings in different geographical contexts and was associated with health behaviors and well-being. Second, educators engaged with mobile applications, wearable devices, AI-driven technologies, telehealth, and online programs. Third, educators’ engagement with these technologies was shaped by individual, technological, organizational, and contextual factors, including leadership support, privacy concerns, time pressure, and workplace culture. **Conclusions:** This review provides a comprehensive synthesis of educators’ digital health, covering digital health literacy, experiences with digital health technologies, and factors influencing engagement. The findings highlight uneven digital health literacy, varied experiences with digital technologies, and the importance of institutional support. They also underscore the need for targeted capacity-building initiatives, context-sensitive interventions, and further research to strengthen educators’ digital health.

## 1. Background

Due to the rapid development of digital technologies, an increasing number of people are using these tools to access health information, monitor their health status, and engage in preventive and self-management practices. Digital health refers to the use of digital technologies to support health management, monitoring, and other health-related practices or services [1,2]. Within this review, digital health represents the broader domain of health-related activities supported by digital technologies, while digital health technologies refer to the technological tools through which these activities are undertaken. These technologies include mobile health applications, wearable devices, telehealth services, online intervention programs, and artificial intelligence (AI). In the context of educators, digital health includes both personal and occupational or professional use of digital technologies, ranging from managing and monitoring educators’ own health and well-being (e.g., monitoring health status, obtaining health information, and supporting self-care) to addressing health needs related to their work (e.g., voice health and work-related stress). These technologies may be used independently by educators or provided as workplace-supported interventions, such as school-based well-being programs. Digital health technologies have the potential to improve health-related outcomes, enhance healthcare access, increase health knowledge, and promote engagement in health behaviors [3,4,5,6]. However, it also raises concerns about information reliability, data privacy, and unequal access to digital resources [7,8,9], highlighting the importance of individuals’ ability to effectively engage with digital health technologies, as well as the need for social and institutional support to facilitate access and use.

Recently, an increasing number of digital health technologies and interventions have been developed for educators [10,11,12]. Educators face a range of health challenges, such as voice disorders, musculoskeletal pain, and visual disorders [13,14,15]. In addition, high work-related stress, emotional demands, and workload are associated with mental health issues, including burnout and psychological disorders [16,17,18]. These health issues can negatively affect teaching effectiveness, job satisfaction, and educational outcomes, as well as lead to increased absenteeism and staff turnover. However, many educators may neglect their own health, delay self-care, and lack time or energy to attend to their well-being due to heavy workloads, time pressure, emotional demands, and conflicting responsibilities [15,19,20,21]. Therefore, digital health technologies may serve as useful tools to help educators monitor their health status, receive timely feedback, and access appropriate support. However, effective engagement with these technologies requires sufficient digital health literacy, which, in the context of educators, refers to their ability to access, evaluate, understand, and appropriately apply health information and services obtained through digital technologies. This includes the ability to identify disinformation and misinformation, protect personal data and privacy, understand and apply health guidance, and communicate effectively with digital systems. For conceptual clarity, this review distinguishes between digital health literacy and competence, on the one hand, and digital health technology use, on the other. Digital health literacy and digital health competence are treated as closely related and substantially overlapping concepts that concern individuals’ ability to engage with digital health information and technologies effectively and appropriately. These abilities may include judging the reliability of digital health information, selecting appropriate technologies for specific health-related purposes, protecting personal data and privacy, and appropriately applying health guidance. In contrast, digital health technology use refers to educators’ actual use or engagement with digital health technologies, without implying a particular level of proficiency, effectiveness, or appropriateness. Thus, literacy and competence concern an individual’s capacity to engage with digital health, whereas technology use concerns the actual behavior or experience of using digital health technologies.

Within the digital health ecosystem, educators occupy multiple and overlapping roles. They are users and recipients of digital health technologies and information for their own personal and occupational health, while also serving as intermediaries who communicate health information and model health-related digital practices within educational settings. Their engagement with digital health is therefore situated within broader technological and organizational conditions, including the accessibility and characteristics of digital technologies, institutional support and guidance, workload, and privacy considerations. Educators’ digital health engagement therefore reflects an interaction between individual competencies and the technological and organizational conditions in which digital health technologies are accessed and used. These roles make educators’ health literacy and digital health literacy important not only for supporting their own health management, but also for their professional roles, as educators’ digital health literacy can influence students’ digital health literacy, which is essential for developing future digital citizens [22,23]. This is because students spend a significant amount of time in school and interact closely with educators, who play a central role in promoting health in schools and shaping students’ health-related knowledge and behaviors through both formal instruction and everyday interactions. Their attitudes and ability to engage with digital health may influence how health-related concepts are communicated within educational settings. Consequently, limited digital health literacy may hinder educators’ ability to use digital tools effectively and increase the risk of disseminating inaccurate health information.

However, research on educators’ digital health remains limited despite the importance of this topic. Existing reviews related to digital health have primarily focused on specific populations, such as healthcare students [24], health workers [25], office workers [26], and patients [27]. Although these reviews provide important insights into digital health, they generally focus on specific health outcomes or intervention types rather than providing a comprehensive synthesis of digital health engagement among educators.

Beyond these existing reviews, primary studies have examined various aspects of educators’ digital health, including digital health literacy, experiences with digital health technologies, and interventions that support digital health development among educators. However, the existing evidence remains fragmented across different educational settings, geographical regions, and forms of digital health engagement, making it difficult to obtain a comprehensive understanding of educators’ digital health. Given the emerging and heterogeneous nature of this literature, a scoping review is needed to map the breadth of current evidence and identify gaps in knowledge.

### Objectives

Educators are increasingly engaging with digital health technologies for personal and occupational health purposes, yet the evidence remains fragmented across educational contexts, technologies, and research domains. Accordingly, this scoping review aims to map and synthesize the existing literature on educators’ digital health and identify areas requiring further investigation.

Specifically, the review addresses three objectives: (1) to examine educators’ digital health literacy and related competencies; (2) to explore educators’ experiences with digital health technologies; and (3) to identify factors that influence educators’ capacity to engage with and navigate the digital health ecosystem.

## 2. Methods

Following the five-stage scoping review framework proposed by Arksey & O’Malley [28], this scoping review was conducted through five stages: (1) identifying the research question, (2) identifying relevant studies, (3) study selection, (4) charting the data, and (5) collating, summarizing and reporting the results. This scoping review was not preregistered, and no publicly accessible review protocol was available.

*Stage 1: Identifying the research questions/objectives*

The research questions of this study are: (1) What is known about the status of educators’ digital health literacy and related competencies, and what factors are associated with them. (2) What are educators’ experiences with digital health technologies. (3) What factors influence educators’ capacity to engage with and navigate the digital health ecosystem?

*Stage 2: Identifying relevant studies*

**Figure 1:**
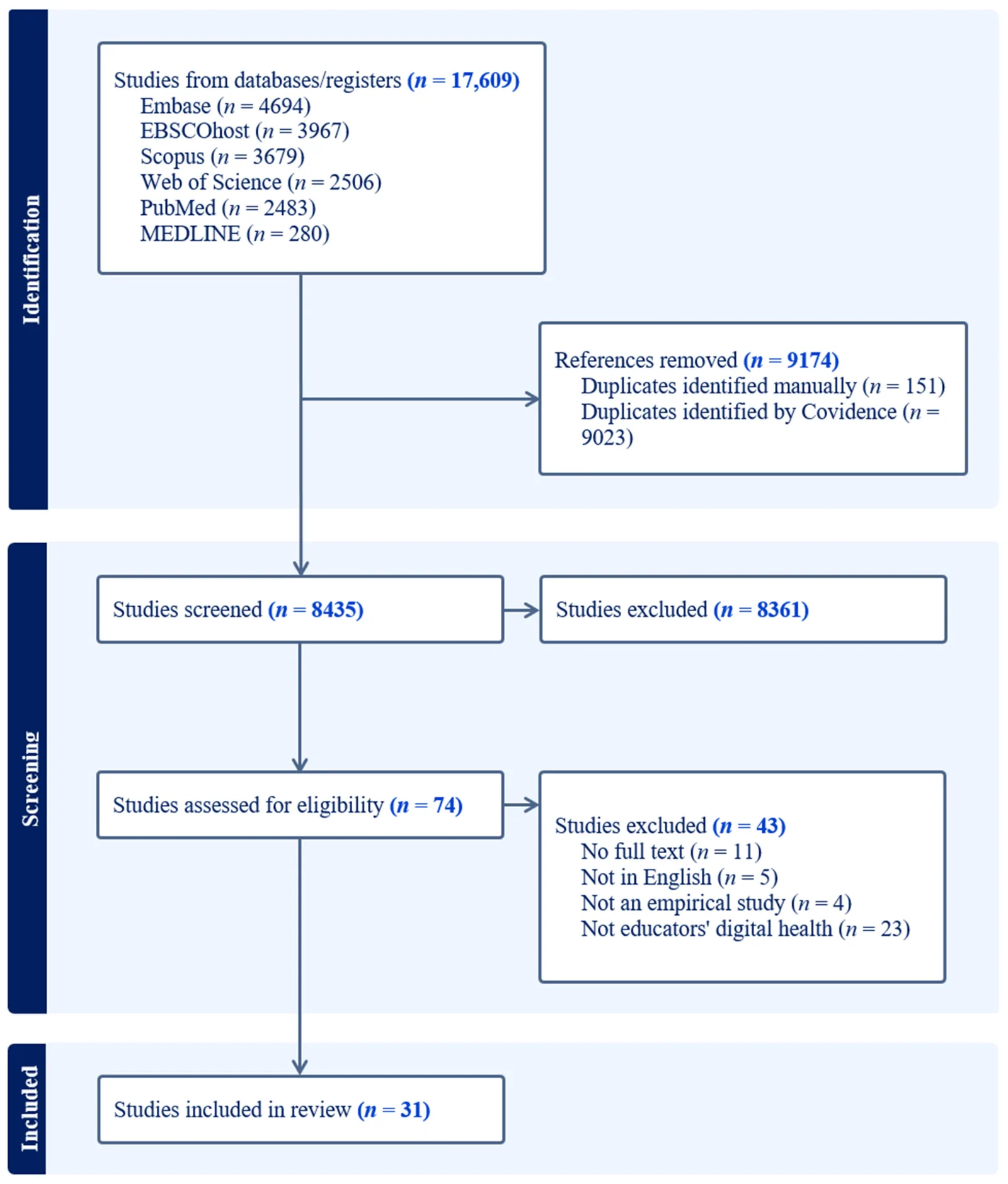
PRISMA 2020 flow diagram [31] of study identification, screening, eligibility assessment, and inclusion for this scoping review of digital health among educators. The figure shows the number of records identified through databases searching, duplicates removed, records screened, full-text articles assessed for eligibility, and studies included in the final review. Searches were conducted on 8 June 2026.

This scoping review was reported in accordance with the PRISMA-ScR guidelines [29] and the PRISMA-S extension for reporting literature searches [30]. We conducted database searches in Scopus, Web of Science Core Collection, PubMed, Embase, and multiple databases accessed via EBSCOhost, including APA PsycInfo, CINAHL, Education Source Ultimate, and ERIC, to identify relevant studies. The complete list of databases searched and all search strings are provided in the Supplementary Material. No study registries (e.g., OSF Registries), online or print sources (e.g., journal websites or conference proceedings), contact with authors or experts, or additional search methods were used in this review. A PRISMA flow diagram was used to show the process of literature search and data charting (Figure 1). The final database searches were conducted on 8 June 2026 and included studies available up to that date. Search strategies were developed using combinations of keywords related to educators and digital health concepts. No search strategies from previous literature reviews were adapted or reused in this review. Synonyms and related terms were combined using the Boolean operator OR, while the two concept groups were combined using the Boolean operator AND. Truncation symbols (*) were used where appropriate to capture variations in keywords. The educator-related terms included educator *, teacher *, “academic staff”, instructor *, professor *, lecturer *, tutor *, “faculty member *”, “teaching staff”, “teacher educator *”, “school staff”, “education personnel”, and “academic personnel”. The digital health-related terms included “digital health”, ehealth *, “e-health”, “e health”, mhealth*, “m-health”, “mobile health”, telehealth *, telemedicine *, “digital health literacy”, “ehealth literacy”, “e-health literacy”, “e health literacy”, “health app *”, “mobile health app *”, “wearable *” W/3 health, and “fitness tracker *”. Controlled vocabularies (e.g., MeSH terms in PubMed and EMTREE in Embase) such as faculty, teacher, school teachers, digital health, telemedicine, wearable electronic devices, mobile applications, and health literacy were used where applicable. Search was conducted using database-specific field modifiers in the title, abstract, and keyword fields where applicable. No limits or restrictions (e.g., publication date, language, or study design filters) were applied during the database searches, and no published search filters were used. Language eligibility was subsequently assessed during study selection, and only empirical, peer-reviewed articles published in English were included. No search update methods (e.g., rerunning searches or email alerts) were used in this review. The search strategy was discussed among the review team during its development. Backward and forward citation searching of included studies was conducted to identify potentially relevant publications. Although citation searching identified several additional records, these records were either duplicates of studies retrieved through the database searches or could not be assessed because the full text was unavailable. Therefore, no additional studies were included.

During the database search, 17,609 records with related topics were identified. Duplicate records were identified and removed using Covidence (Veritas Health Innovation, Melbourne, Australia; https://www.covidence.org/), followed by manual verification by the reviewers. After removing 9174 duplicated items, 8435 items remained for further screening (Figure 1).

*Stage 3: Study selection*

We used a set of criteria to ensure that only full-text, peer-reviewed articles were included in this study. Table 1 shows the inclusion and exclusion criteria adopted in this study.

### 2.1. Participants

Studies were included if they involved educators. Educators referred to teaching professionals working in educational settings, such as school teachers, university instructors, tutors, and other teaching personnel. Studies focusing exclusively on students, healthcare professionals, or non-educational occupations were excluded. This definition was applied consistently as an eligibility criterion during study selection.

### 2.2. Concept

Studies were included if they examined issues related to educators’ digital health, which refers to the use of digital technologies to support health management, monitoring, and healthy practices among educators. Studies unrelated to educators’ digital health were excluded.

### 2.3. Context

The review considered studies conducted in any educational setting or geographic region. No restrictions were applied regarding region, culture, or educational setting.

### 2.4. Sources of Evidence

The review included only empirical, peer-reviewed studies published in English. Due to resource and feasibility constraints, studies published in other languages were excluded. Non-empirical publications and grey literature sources, such as reviews, editorials, commentaries, dissertations, policy documents, and reports, as well as non-peer-reviewed articles, were excluded to focus the review on empirical evidence published in peer-reviewed journals. Conference proceedings were eligible if they had undergone peer review and met all inclusion criteria.

According to the inclusion and exclusion criteria, we screened 8435 items that remained after removing the duplicates. Title and Abstract screening led to the exclusion of 8361 items, with 74 remaining. Scope of research criterion led to the exclusion of 4 items, written language criterion led to the exclusion of 5 items, and research topic criterion led to the exclusion of 23 items, and no accessibility to the full text led to the exclusion of 11 items. As a result, 43 items were excluded, and 31 articles were included in the review. The entire process of selecting the relevant studies is detailed in the PRISMA flow diagram in Figure 1. The same two reviewers independently conducted title and abstract screening and full-text screening using Covidence. Disagreements regarding study eligibility were discussed and resolved through consensus. Prior to screening, the inclusion criteria were discussed, and screening decisions were periodically reviewed throughout the process.

*Stage 4: Charting the data*

Data charting was conducted using Covidence. A standardized data extraction form was developed based on the Covidence template and adapted to align with the objectives of this review. The form was piloted on two to three studies prior to full data charting and subsequently refined during the early stages of extraction as needed to improve clarity and consistency. Data charting was performed by one reviewer. The reviewer subsequently re-checked the extracted data for accuracy and consistency, and a second reviewer checked the charted data for errors and inconsistencies. No formal critical appraisal of the methodological quality or risk of bias of the included studies was conducted, as this scoping review aimed to map and synthesize the existing literature on educators’ digital health and identify gaps in current knowledge rather than assess the methodological quality or certainty of the evidence.

For each selected article, the following information was extracted: author(s), year of publication, region, schools that participants worked in (educational setting), research question/aim, study design, sample size, data collection methods, and findings. All information was presented in Table 2, Table 3 and Table 4.

*Stage 5: Collating, summarizing and reporting the results*

To present a narrative account of the previous literature, an analytic framework or thematic construction was required [28]. The extracted data were summarized into two parts. First, descriptive information (e.g., authors, publication years, regions, sample size, and educational setting, study design, and data collection methods) was charted to provide an overview of the 31 studies. Then, three themes of concern were identified in the 31 articles in this study:

1. Educators’ digital health literacy and related competencies.
2. Educators’ experiences with digital health technologies.
3. Factors influencing educators’ capacity to navigate the digital health ecosystem.

The findings were synthesized thematically according to these three themes, which corresponded to the three research questions of the review. Given the heterogeneity of the included studies, the synthesis focused on recurring patterns, differences, and gaps across the literature rather than direct comparisons of outcomes across studies.

## 3. Results

### 3.1. Characteristics of Sources of Evidence

A total of 31 empirical studies published in peer-reviewed journals were identified and reviewed. The studies were published between 2015 and 2026, with most published from 2021 onward. Geographically, the studies were concentrated in Europe and Asia, with comparatively fewer studies from Africa, Oceania, North America, and South America.

**Figure 2:**
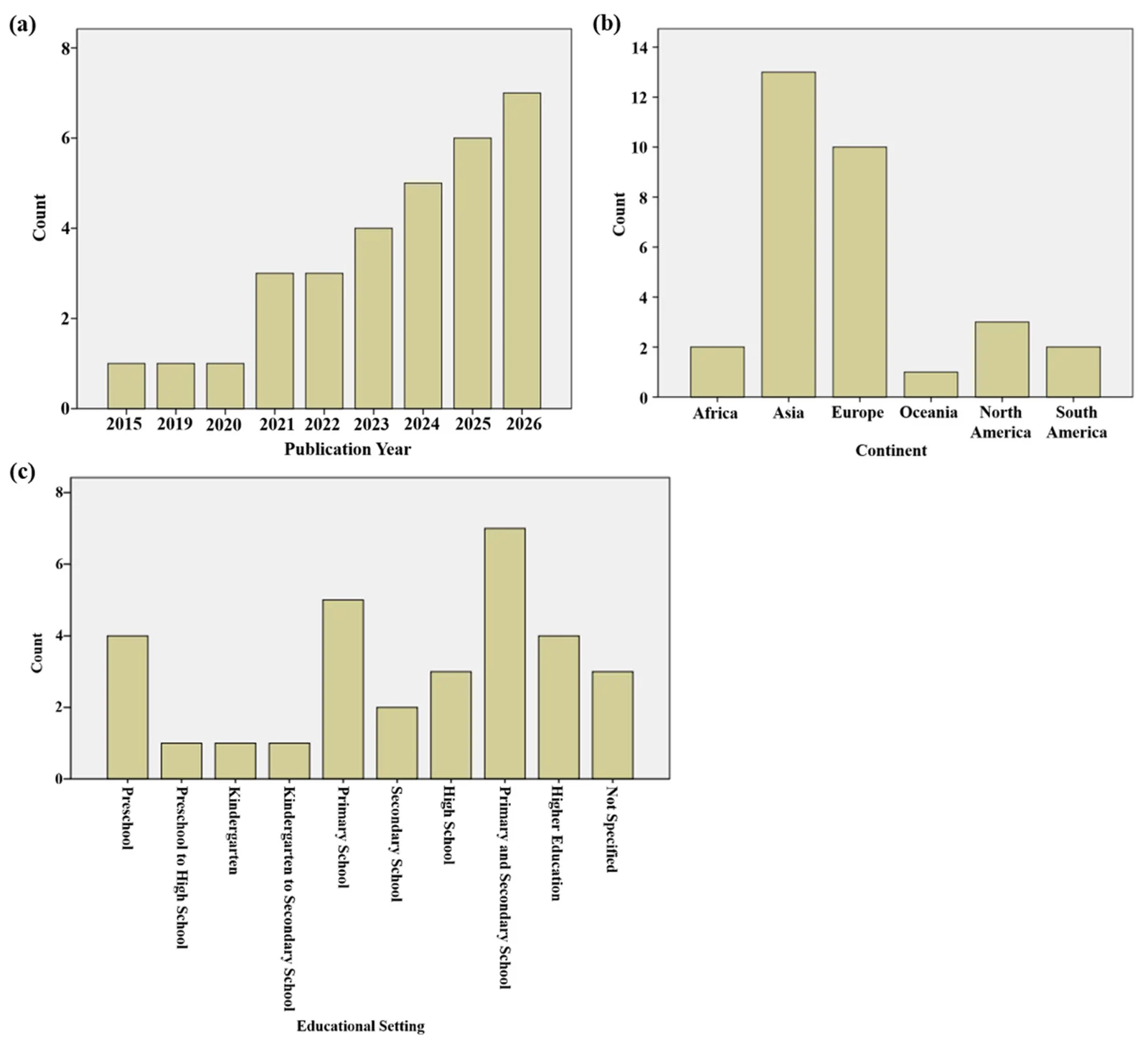
Descriptive characteristics of the studies included in this scoping review of digital health among educators: (**a**) shows the distribution of included articles by publication year, (**b**) shows the geographic distribution of studies by continent, and (**c**) shows the educational settings in which the studies were conducted.

The included studies represented a wide range of educational settings, including preschool education, kindergarten education, primary and secondary education, and higher education contexts. However, most studies focused mainly on primary and secondary school teachers, indicating comparatively limited attention to educators in other educational settings. Sample sizes ranged from 8 [43,61] to 3375 [39], suggesting substantial variation in study scope and design (Table 2; Figure 2).

Regarding methodology, most studies adopted quantitative designs (*n* = 23), while fewer studies adopted qualitative (*n* = 7) or mixed-methods (*n* = 1) designs. The included studies also used various data collection methods and evidence sources, with surveys being the most common method (*n* = 25), followed by physiological evaluations (*n* = 7), interviews (*n* = 6), app log data (*n* = 1), audio recording (*n* = 1), clinical evaluation (*n* = 1), empirical taxonomy-development study using a systematic review as one data source (*n* = 1), observation (*n* = 1), and visual mind mapping (*n* = 1). This distribution suggests a predominance of self-reported data within the current evidence base. The details of the data collection methods used in the reviewed studies were shown in Table 3.

### 3.2. Synthesis of Results

#### 3.2.1. Educators’ Digital Health Literacy and Related Competencies

Across the reviewed studies included in this theme, educators’ digital health literacy and related competencies were examined. The included studies used both “digital health literacy” and “eHealth literacy” terminology. For this review, eHealth literacy was treated as a closely related construct within the broader domain of digital health literacy because both concern individuals’ ability to engage with health information and services through digital technologies. However, these terms were not assumed to be conceptually or psychometrically identical, and the included studies used heterogeneous measures and operationalizations of these related constructs. Accordingly, findings in this theme were synthesized thematically rather than treated as directly comparable across studies. Among Turkish preschool teachers, Çelik Çoban and Büyükdereli Atadag [32] reported a moderate level of digital health literacy, with the highest scores in the “Adding Content” subscale and the lowest scores in the “Protecting Privacy” subscale. Khancolabi et al. [33] reported that Iranian primary school teachers had a mean digital health literacy score below the standard level. Among Turkish higher education teachers, Koştu and Arslan [34] reported moderate levels of digital health literacy and AI literacy. Among Australian primary and secondary school pre-service teachers, Peralta et al. [35] reported high digital health literacy across most domains, while only moderate levels were found in the “Feeling safe and in control”, “Access to digital services that work”, and “Digital services that suit individual needs” subscales. Among German primary and secondary school teachers, Rangnow et al. [36] showed that the greatest challenges in using digital health information were protecting privacy and evaluating reliability. Together, these studies suggested considerable variability in reported levels of educators’ digital health literacy and related competencies across educational settings and regional contexts. Despite differences in the constructs and measures assessed, the studies identified challenges across multiple domains, including protecting privacy, evaluating the reliability of digital health information, and feeling safe and in control when using digital services.

In addition, several studies investigated factors associated with digital health literacy, including demographic, health-related, behavioral, and professional factors. Specifically, Çelik Çoban and Büyükdereli Atadag [32] demonstrated that higher digital health literacy was associated with higher infodemic awareness, which is the ability to recognize misinformation and information overload. Khancolabi et al. [33] showed that digital health literacy was positively predicted by health concerns, daily Internet use, regular physical activity, general health knowledge, and the use of health information via social networks. Koştu and Arslan [34] showed that digital health literacy positively predicted healthy lifestyle behaviors and that it had a stronger and more stable association with healthy lifestyle behaviors than AI literacy. Peralta et al. [35] demonstrated that pre-service teachers in Tasmania felt less socially supported and less understood by healthcare professionals than those in New South Wales, but they reported feeling safer and more in control when accessing digital health services. Rangnow et al. [36] showed that female teachers and those in leadership positions more often reported sufficient ability to communicate health information, whereas male teachers more often reported sufficient ability to protect their privacy. Among Turkish teachers, Demir et al. [38] showed that teachers aged 30–39 and those with 10–19 years of teaching experience had higher digital health literacy than other groups. In addition, they showed that higher digital health literacy was correlated with higher cyberchondria, which is health-related anxiety resulting from excessive online health searches. Among Chinese kindergarten to secondary school teachers, Feng et al. [39] found that higher digital health literacy was associated with greater cognition of cancer warning symptoms and served as a mediator with a partial masking effect in the relationship between self-efficacy and cancer cognition. Among Taiwanese K-12 teachers, Huang et al. [40] found that teachers aged 23–31 had higher digital health literacy than those aged 42–51. They also found that higher functional digital health literacy was correlated with better medication adherence. Among Portuguese early childhood education teachers, Rosário et al. [41] showed that higher digital health literacy was associated with better well-being, with teacher–child interaction partially mediating this relationship. Because the mediation analyses reported by Feng et al. [39] and Rosário et al. [41] used cross-sectional survey data, these mediation findings indicate statistical associations and do not establish temporal or causal relationships among the variables. Together, these studies suggested that educators’ digital health literacy was associated with a wide range of demographic, behavioral, health-related, and professional factors, as well as positive health- and work-related outcomes, across different regions and educational settings.

Only one study proposed a conceptual framework of digital health literacy for educators. Specifically, Yang and Shi [37] constructed a framework of digital health literacy for primary and secondary school physical education teachers, including three core dimensions: digital health awareness and critical literacy, digital teaching practice and innovative application competence, and digital security ethics and environmental awareness competence.

**Figure 3:**
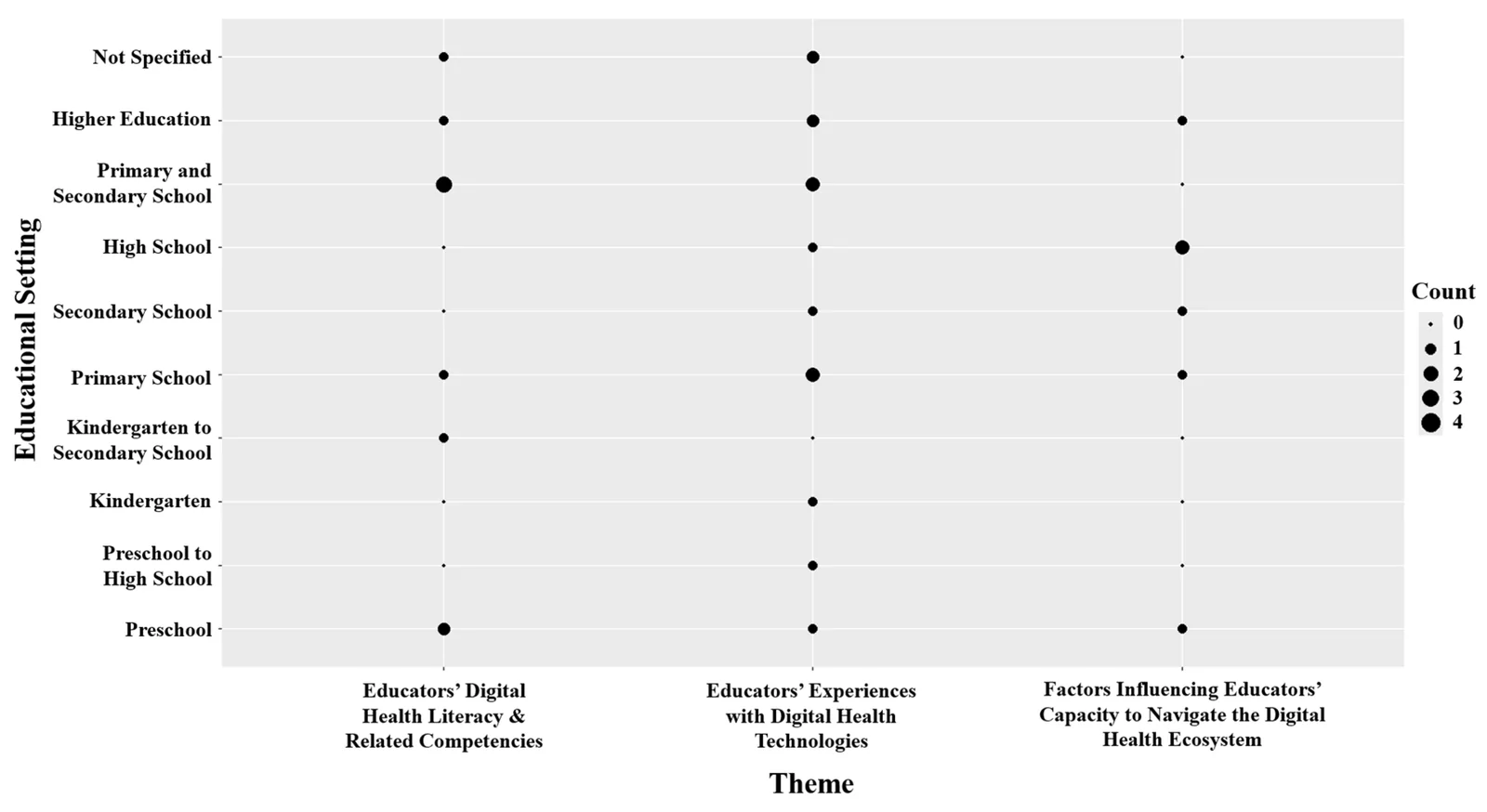
Evidence gap map of the studies included in this scoping review of digital health among educators. The figure shows the distribution of reviewed studies across the three identified themes (i.e., educators’ digital health literacy and related competencies, educators’ experiences with digital health technologies, and factors influencing educators’ capacity to navigate the digital health ecosystem) and different educational settings. Bubble size represents the number of study occurrences within each theme and educational setting category, highlighting areas with limited or absent evidence. One included study was relevant to two themes and was therefore counted in both categories, resulting in 32 study occurrences across 31 unique studies.

Together, the reviewed studies covered various educational settings, ranging from early childhood education to higher education, although most studies focused on primary and secondary school teachers. Three studies focused on preschool or kindergarten teachers, and only one study investigated higher education teachers. In addition, one study did not clearly specify the educational setting of the participants. Geographically, the reviewed studies under this theme were conducted primarily in Asia and Europe, with only one study conducted in Oceania, and no eligible studies were identified from Africa, North America, or South America. Methodologically, most reviewed studies relied on survey-based data collection methods, suggesting a strong reliance on self-reported evidence within the current literature (Table 4; Figure 3).

#### 3.2.2. Educators’ Experiences with Digital Health Technologies

Several reviewed studies examined educators’ experiences with multiple types of digital health technologies. For instance, Manning et al. [56] reported that 12 out of 14 high school teachers in the United Kingdom used digital technologies, including applications and wearable devices, to self-manage their physical or psychological well-being. They also reported using wearable devices in the workplace and applications outside of school. Manning et al. [43] found that digital health technologies helped teachers recognize the physical and psychological toll of their work, thereby fostering self-compassion, empathy toward colleagues, and stress management. As teachers returned to school following COVID-19 lockdowns, they demonstrated more intentional self-care, even without referring to the technologies. Kwon et al. [42] developed, implemented, and evaluated a multitiered intervention to improve the well-being of preschool teachers in the United States. The intervention consisted of four key elements: a wearable fitness tracker, ten weekly self-guided online modules, individualized wellness coaching, and environmental and center-level supports. They found that the intervention significantly improved teachers’ physical, psychological, and professional well-being. Together, these studies suggested that multiple types of digital health technologies could support educators’ physical, psychological, and professional well-being by promoting self-awareness, self-care, stress management, and healthy workplace practices.

Several reviewed studies examined educators’ experiences with mobile health and smartphone applications. In particular, Hauck et al. [44] evaluated the mobile application *ReSt* for improving voice health among German primary and secondary school teachers. Compared with the control group, teachers who received the app-based voice training showed improvements in objectively measured voice capability. Spending more time on average exercising in the app was associated with a greater increase in self-reported voice health, but no clear differences between the control and training groups were found. Overall, participants reported acceptable-to-positive usability for the application. Hirshberg et al. [45] investigated the effects of the Health Minds Program, a smartphone-based meditation intervention for well-being, among preschool to high school teachers in the United States. The training group demonstrated larger reductions in psychological distress over the intervention period compared with the control group. In addition, participants in the training group showed increased overall well-being, mindful action, self-compassion, cognitive defusion, and meaning in life, as well as decreased loneliness and perseverative thinking. The intervention also predicted lower odds of experiencing a categorical increase in anxiety or depressive symptoms. Scanferla et al. [46] tested the usability and acceptability of the mHealth application “Health and Voice”, designed to support vocal health among primary school teachers in Brazil. They found that the application demonstrated high usability and acceptability. In addition, teachers’ self-perceived knowledge of vocal care increased after using the application. Together, these studies suggested that mobile health and smartphone applications can effectively support educators’ well-being and voice health through accessible, acceptable, and user-friendly digital interventions.

Several reviewed studies examined educators’ experiences with wearable devices. For example, Klatt et al. [47] investigated the use of wrist-worn fitness trackers to measure German teachers’ heart rate as a physiological indicator of stress during teaching. They found that the fitness tracker successfully mapped the physiological stress response: heart rate significantly increased before the micro-teaching unit, peaked during the micro-teaching unit, and decreased afterward. Shirouzu et al. [48] used a wearable device that measured ECG and acceleration to objectively assess stress among Japanese kindergarten teachers. They found that the device successfully identified teachers experiencing high levels of stress by monitoring peaks in sympathetic nervous system activity during working hours. In addition, one teacher who exhibited less polite language and behavior was found to have severe sleep apnea and autonomic nervous system activity levels comparable to those of patients with clinical depression or adjustment disorder. Together, these studies suggested the potential of wearable devices to provide objective, real-time assessments of educators’ physiological stress responses, supporting the identification and monitoring of work-related stress.

Several reviewed studies examined educators’ experiences with AI-driven technologies. Specifically, Jalani et al. [49] evaluated an AI-enabled workplace health kiosk among teachers in the Philippines. They found that the kiosk had high user acceptability and usability. After 1 to 2 weeks of use, 70.9% of participants had consulted a health care professional, and 66.7% reported making lifestyle changes. Tachie-Donkor et al. [50] explored the use of AI-driven technologies to promote health and well-being among higher education teachers in Ghana. They showed that AI-driven technologies could positively influence health behaviors, encourage proactive adoption of healthier lifestyles, and improve well-being. In addition, participants perceived that these technologies could provide early warnings of potential health complications, although they emphasized that the information should be confirmed by a medical practitioner. Participants also expressed concerns about privacy, potential health risks, and psychological consequences of continuous self-monitoring using AI-driven technologies. Together, these studies suggested that AI technologies have the potential to support educators’ health and well-being by promoting healthier behaviors and facilitating early health awareness, although concerns regarding privacy, potential health risks, and the need for professional medical confirmation remain.

Several reviewed studies examined educators’ experiences with telehealth interventions. For instance, Grillo [51] examined the effects of the telepractice Global Voice Prevention and Therapy Model on pre-service teachers in the United States. The results showed that the intervention significantly improved participants’ vocal resonance and pitch stability, while reducing the negative impact on voice-related quality of life and decreasing vocal fatigue during teaching. Kayabınar et al. [52] investigated the effects of a preventive telerehabilitation program for musculoskeletal problems among primary and secondary school teachers in Turkey. They showed that the intervention significantly improved participants’ musculoskeletal health. Liu et al. [53] investigated the effects of a telepractice voice intervention among female primary school teachers in China. They showed that the intervention improved objective voice parameters, including acoustic and aerodynamic measures, as well as clinician-based perceptual ratings. Saintila et al. [54] investigated the effects of a telehealth program on nutritional knowledge and body mass index (BMI) among higher education teachers in Peru. They found that the program significantly reduced teachers’ weight and BMI while increasing their basic nutritional knowledge. Together, these studies suggested that telehealth interventions can effectively improve educators’ health across multiple domains, including vocal health, musculoskeletal health, nutritional knowledge, and physical health outcomes.

Only one reviewed study examined educators’ experiences with an online program. Specifically, Vidal-Conti and Galmes-Panades [55] investigated the effect of an online postural education program on Spanish primary school teachers’ knowledge of back care and low back pain prevention. Teachers in the experimental group demonstrated increased knowledge of low back pain prevention after the intervention as compared with baseline levels, with greater improvement than the control group. This study suggested that online educational programs can effectively improve educators’ knowledge of health-related topics and support preventive health education.

Together, the reviewed studies covered various educational settings, ranging from preschool to higher education, although most studies focused on primary and secondary school teachers. Methodologically, the reviewed studies relied not only on self-reported data collected through surveys and interviews, but also on objective measures, including physiological assessments, clinician evaluations, and application usage logs. The reviewed studies examined educators’ experiences with various digital health technologies, including mobile applications, wearable devices, AI-driven technologies, telehealth interventions, and online programs. They focused primarily on stress management, well-being, voice health, musculoskeletal health, back pain prevention, and healthy lifestyles (Table 4; Figure 3).

#### 3.2.3. Factors Influencing Educators’ Capacity to Navigate the Digital Health Ecosystem

Several reviewed studies examined facilitators of educators’ capacity to navigate the digital health ecosystem. For instance, Hsu et al. [57] demonstrated that online learning communities facilitated mental health literacy by providing supportive digital spaces for Taiwanese preschool teachers to share experiences and access empathic support from peers. MacIsaac et al. [58] used mind mapping to explore Scottish secondary school teachers’ engagement with digital health technologies. They found that teachers interacted with these technologies in complex and multifaceted ways, highlighting opportunities for developing more critical forms of engagement. The authors further suggested that mind mapping may support more critical and reflective engagement. Manning et al. [56] showed that digital well-being interventions endorsed by school leaders were perceived as more credible and were more likely to be adopted by teachers. Manning et al. [59] developed a taxonomy framework to support the systematic selection of digital companions for high school teachers’ stress self-management in the United Kingdom. The framework included classifications of stress management strategies (educational, physiological, cognitive, and social) and categories of digital techniques according to their mechanisms of action, including fostering reflection, suggesting treatment, peer support, and entertainment. The authors also proposed selection criteria for digital companions, including suitability, availability, evaluation, security, validity, and cost. Manning et al. [61] showed that features such as passive data collation, brief and non-intrusive interactions, reminders, and clear data visualization supported teachers’ engagement with digital health technologies. In addition, school leadership could help normalize the use of such technologies to support teachers’ well-being. Musakuro and Gie [62] identified seven key factors that contributed to a positive “experience gained” among higher education teachers in South Africa: perceived usefulness of e-health tools, perceived usefulness of social media, user experience and satisfaction, university commitment, variety of digital mental health services, post-COVID-19 delivery mode preference, and ethical considerations. Yin et al. [60] constructed a health literacy index system for Chinese primary school teachers in rural regions and showed that digital health empowerment had a direct positive effect on teachers’ health literacy, highlighting its potential role in supporting health-related competencies. Together, these studies suggested that educators’ capacity to navigate the digital health ecosystem can be strengthened through supportive organizational environments, thoughtfully designed digital technologies, and interventions that promote digital health literacy, critical engagement, and user empowerment.

In addition, several reviewed studies examined barriers to educators’ capacity to navigate the digital health ecosystem. For example, MacIsaac et al. [58] showed that negative emotional experiences influenced and potentially transformed teachers’ interactions with digital health technologies. Manning et al. [56] showed that teachers consistently acknowledged the presence of workplace stress, highlighting both physical and cultural factors that influenced their ability to manage stress. Manning et al. [61] identified limited guidance on features, connectivity issues, demands for extended interaction, and the inability to distinguish between different activity data as barriers to educators’ engagement with digital health technologies. In addition, contextual constraints including time pressure, social stigma, and a lack of private space for stress management after returning to school further limited engagement. Musakuro and Gie [62] reported that teachers were concerned about the absence of a legal framework for digital health services during COVID-19. Together, these studies suggested that educators’ capacity to navigate the digital health ecosystem can be hindered by psychological, organizational, technological, and regulatory barriers that limit engagement with digital health technologies.

Together, the reviewed studies identified both facilitators of and barriers to educators’ capacity to navigate the digital health ecosystem. Geographically, the reviewed studies under this theme primarily focused on European, Asian, and African educators. Methodologically, the reviewed studies used various data collection methods, including surveys, visual mind mapping, audio recordings, qualitative data obtained from a previous study, systematic reviews, expert taxonomy development, expert consultation, and physiological evaluation. The reviewed studies showed that facilitators and barriers operate at individual, technological, organizational, and systemic levels, highlighting the importance of supportive environments, user-centered technology design, digital health empowerment, and appropriate institutional and regulatory support for strengthening educators’ capacity to navigate the digital health ecosystem (Table 4; Figure 3).

## 4. Discussion

This scoping review aimed to examine educators’ digital health literacy and related competencies, explore educators’ experiences with digital health technologies, and examine the factors influencing educators’ capacity to engage with and navigate the digital health ecosystem. Across the 31 included studies, the findings revealed substantial variation in educators’ digital health literacy across educational and geographical contexts and identified multiple demographic, health-related, behavioral, and professional factors associated with digital health literacy. The reviewed studies also suggested that educators generally experienced a range of digital health technologies, including mobile applications, wearable devices, AI-driven technologies, telehealth interventions, and online programs, as potentially beneficial for supporting health and well-being, although the technologies examined, outcomes assessed, and reported benefits varied across contexts. In addition, the findings highlighted multiple facilitators of and barriers to educators’ capacity to engage with and navigate the digital health ecosystem. Together, these studies identified individual digital health literacy, experiences with digital health technologies, and organizational and contextual factors as relevant dimensions of educators’ digital health.

### 4.1. Inequality of Digital Health Literacy Among Educators

The reviewed studies suggested substantial variation in educators’ digital health literacy across the educational and geographical contexts represented in the evidence base. Differences were observed across demographic characteristics, including age [38,40], teaching experience [38], leadership roles [36], and gender [36], as well as regional contexts [33,36,38,39,40,41]. In addition, several studies identified associations between digital health literacy and health-related factors, including infodemic awareness [32], health-related behaviors [33,34], medication adherence [40], cancer cognition [39], cyberchondria severity [38], and well-being [41]. Together, these findings indicate reported associations between digital health literacy and a range of demographic, behavioral, health-related, and professional variables.

These patterns were consistent with previous research conducted among general populations in different regional contexts, which had similarly demonstrated disparities in digital health literacy according to sociodemographic characteristics [63], geographic backgrounds [64,65], and educational settings [66]. Therefore, the variation identified across educator groups in this review may reflect broader structural and contextual differences in digital health engagement rather than solely individual technological skills.

The observation of variation in educators’ digital health literacy across demographic and contextual groups suggested potential disparities in educators’ ability to effectively engage with digital health technologies. These findings underscored the need for more targeted and contextually responsive support rather than assuming a one-size-fits-all approach to digital health promotion among educators. Such support strategies may need to be tailored to the specific needs of different educator groups based on their characteristics and working environments. Particular attention may be needed for competencies related to evaluating the reliability of digital health information, protecting personal privacy, and appropriately applying digital health guidance, which were identified as common challenges across the reviewed studies. Practical approaches may include professional development workshops on digital health, school-based training on privacy protection and misinformation identification, and clearer institutional guidance regarding the appropriate use of digital health technologies. In addition, further research is needed to better understand patterns of similarity and difference across educator subgroups and educational contexts to inform the development of more precise and impactful digital health promotion strategies.

The finding also suggested that educators’ digital health literacy should not be considered in isolation from their experiences with digital health technologies. Competencies such as evaluating the reliability of digital health information, protecting privacy, and feeling safe and in control may be relevant to educators’ confidence in adopting and using digital health technologies. Although the reviewed studies did not directly examine these relationships, the findings collectively suggest that variation in digital health literacy may be associated with differences in educators’ engagement with and experiences of digital health technologies across contexts.

### 4.2. From Experience to Future Support

The reviewed studies reported that educators experienced a range of perceived and observed benefits of digital health technologies in relation to stress management, self-care, well-being, and health-related practices when implemented within supportive organizational environments. Across the included studies, educators generally reported positive experiences with digital health technologies, including increased awareness of health-related behaviors, improved access to mental health support, and enhanced engagement in self-management practices [43,44,50,55,60,62]. These results suggest that digital health technologies may be used not only for health monitoring but also to support health awareness, self-management, and health-related behaviors among educators. Similar benefits of digital health engagement have also been reported across broader digital health literature, particularly in relation to well-being [67,68], self-monitoring [69], and health-related behavior change [70].

Although educators generally reported positive experiences with digital health technologies, the reviewed studies suggested that these experiences were shaped not only by individual motivation and technological features but also by organizational, cultural, and contextual conditions. In particular, the reviewed studies showed institutional endorsement, leadership support, access to digital tools that aligned with educators’ professional needs, and clear practical guidance as facilitators of engagement with digital health technologies [43,56,58,59,61], whereas time pressure, privacy concerns, limited guidance, and workplace stigma were reported as barriers to sustained use [50,56,61,62]. These patterns were consistent with broader digital health literature showing that organizational support, allocated time, privacy protection, and clear guidance are important factors influencing the adoption and continued use of digital technologies [71,72,73]. The findings therefore suggest that successful implementation of digital health technologies in educational settings may require attention not only to technological design, but also to the social and institutional environments in which educators work.

An important feature of the current evidence base is that several studies conducted by Manning and colleagues collectively provided a coherent line of inquiry into educators’ engagement with digital health technologies. Rather than examining isolated aspects of digital health, these studies progressively explored educators’ use of digital health technologies, their experiences and perceptions, the barriers and facilitators influencing sustained engagement, and the development of a taxonomy to support the selection of appropriate digital companions for stress self-management [43,56,59,61]. Together, this body of work offered a more comprehensive understanding of factors relevant to the implementation of digital health technologies to support educators’ well-being. At the same time, the concentration of multiple studies within this single research group should be considered when interpreting the overall evidence base. Although these studies addressed different research questions and used diverse methods and interventions, they were all conducted with educators in UK secondary or high-school contexts. This concentration may therefore give greater representation to the experiences and needs of educators in this particular educational and geographical context, while limiting the extent to which findings are independently replicated across other research teams, educational settings, and geographical contexts. Further research conducted by different research groups and using diverse methodological approaches across educational and geographical contexts would help determine whether these findings are consistently observed more broadly.

Another observation from this scoping review is that digital health was operationalized in diverse forms and for different purposes across the reviewed studies. In terms of form, the included studies examined mobile and smartphone applications, wearable devices, AI-driven technologies, telehealth and telepractice interventions, and online intervention or education programs. In terms of purpose, these technologies were used for personal health and well-being, such as stress management, self-care, health monitoring, and healthy-lifestyle support, as well as for occupational health needs related to educators’ work, such as voice health, musculoskeletal health, postural health, and work-related stress [42,43,44,45,46,47,48,49,50,51,52,53,54,55]. Some studies also focused on providing health information, supporting health-related knowledge and behaviors, or monitoring physiological indicators and identifying potential health concerns [47,48,49,50,51,52,53,54,55]. These categories were not mutually exclusive, as individual technologies could serve multiple health-related purposes and operate across personal and occupational contexts. This diversity indicates that educators’ digital health encompasses a broad range of technological forms, health-related purposes, and implementation contexts. Distinguishing these dimensions may help clarify the boundaries of educators’ digital health and support more precise design and evaluation of future interventions. This categorization also helps contextualize the three themes identified in this review by highlighting how educators’ digital health literacy, experiences with digital health technologies, and the factors influencing their engagement may be considered in relation to the type and purpose of digital health involved.

Across the three themes, the evidence showed both recurring patterns and substantial heterogeneity. Across studies conducted in different educational and geographical contexts, challenges related to protecting privacy and evaluating the reliability of digital health information emerged as recurring areas of difficulty [32,36]. Similarly, leadership and institutional support, clear guidance, and accessible technologies were repeatedly identified as facilitators of digital health engagement [43,56,58,59,61], whereas time pressure, privacy concerns, limited guidance, and workplace barriers were commonly reported as obstacles [50,56,61,62]. However, evidence regarding the effectiveness of digital health technologies was less consistent. Several intervention studies reported improvements in specific health or well-being outcomes [42,45,51,52,53,54,55], whereas other studies primarily examined usability, acceptability, perceived benefits, or associations [43,46,49,50]. For example, Hauck et al. [44] reported improvements in some voice-related measures but no clear differences between the intervention and control groups on some outcomes. These differences suggest that the reported benefits of digital health technologies should be interpreted in relation to the specific technology, outcome, intervention design, and type of evidence available. The evidence base was heterogeneous in terms of study design and data collection methods, participant characteristics, educational setting and context, geographical setting, digital health technologies and purposes examined, and outcomes and measures assessed. Given these differences, findings were interpreted in relation to the specific populations, contexts, technologies, and outcomes examined in each study. The evidence was also predominantly quantitative and survey-based, with substantial reliance on self-reported data.

Together, the reviewed studies suggest that educators’ digital health reflects the interaction of individual competencies, experiences with digital health technologies, and organizational and contextual conditions. Supporting educators’ digital health may therefore benefit from considering these dimensions in combination rather than addressing individual competencies, technology design, or institutional support in isolation.

### 4.3. Limitations

This review has several limitations that should be considered. First, the search was restricted to academic articles published in English, which may have excluded relevant studies published in Chinese or other languages. This language criterion may have affected the geographical representation of the evidence across Asia, Europe, and other regions, particularly in settings where relevant research is more commonly published in local or regional languages. Studies published in local or regional journals may also contain context-specific evidence that was not captured in this review. Future multilingual scoping reviews could help broaden the geographical and contextual representation of evidence on educators’ digital health. Second, the review was restricted to empirical, peer-reviewed articles and therefore excluded grey literature, such as dissertations, reports, policy documents, and other non-peer-reviewed publications. This restriction may have excluded relevant evidence and limited the breadth of perspectives represented in the review. These eligibility restrictions may also have contributed to publication and selection bias in the evidence base, as relevant evidence that was unpublished, not peer-reviewed, or not published in English was not captured.

Third, several included studies were conducted by the same research groups, which may indicate some concentration within the current evidence base and may limit the extent to which findings are independently replicated across research teams. Fourth, the included studies were methodologically heterogeneous in terms of study design, data collection methods, participant characteristics, educational level and context, geographical setting, digital health technologies and purposes examined, and outcomes assessed. They also used heterogeneous definitions and operationalizations of digital health and digital health literacy and related constructs, including eHealth literacy, and employed different measurement instruments. This heterogeneity limits the direct comparability of reported findings across studies and requires the findings to be interpreted in relation to the specific contexts and constructs examined. Fifth, some included studies did not clearly report key information such as participant characteristics. This incomplete reporting limited the completeness of the evidence synthesis and may have restricted comparisons across studies. Sixth, the included studies varied substantially in educational setting, educator population, geographical context, and working environment, with most focusing on primary and secondary school teachers. This variation may limit the transferability of findings across different educator groups and educational contexts. Seventh, although data charting was conducted by one reviewer, the extracted data were re-checked by the same reviewer and subsequently checked by a second reviewer for errors and inconsistencies. However, this approach may still be subject to errors or subjective interpretation compared with independent duplicate data charting. Eighth, the evidence base was predominantly quantitative and survey-based, and several included studies used cross-sectional designs. Many studies relied on self-reported measures. This predominance should be considered when interpreting the findings, as cross-sectional designs limit assessment of temporal or causal relationships, while self-reported data may be subject to recall and response biases and may not fully capture educators’ actual digital health practices or behaviors.

## 5. Conclusions

The findings of this scoping review highlighted the importance of digital health within the context of educators and suggested that access to digital health technologies alone may be insufficient, with institutional, professional, and technological support also potentially relevant to educators’ engagement with these tools. As digital technologies become increasingly integrated into health management and everyday life, the reviewed studies suggest that digital health literacy is associated with aspects of educators’ health and well-being and may also be relevant to their professional roles.

This review further suggests several practical implications for policymakers, health professionals, and researchers. For digital health technology developers, the evidence highlights the importance of designing solutions that are tailored to different educator groups based on their characteristics, needs, and contexts. For school leaders and professional development providers, the results demonstrate the need for institutional support and environments that facilitate educators’ engagement with digital health technologies, enhancing their digital health literacy and overall health and well-being. For researchers, this review highlights the need for further investigation into educators’ digital health, including studies that identify patterns of similarity and difference among educator groups and develop effective digital health promotion strategies. For educators, this review highlights the importance of digital health literacy for engaging with digital health information and technologies in personal and professional contexts.

This review also illuminates several avenues for future research. The fact that few studies have focused on educators’ digital health highlights the need for more attention to this field. Future research should expand to include under-represented populations, such as kindergarten teachers and educators from regions that are currently less studied, to create a more comprehensive understanding across educational settings and contexts. Finally, longitudinal and intervention-based studies are also needed to examine the long-term impact of digital health technologies and interventions on educators’ digital health, well-being, and professional practice.

## Figures and Tables

**Table 1:** Inclusion and exclusion criteria used to select studies for this scoping review of digital health among educators. The table summarizes the eligibility criteria applied to scope of research, type of documents, written language, research topic, and full-text accessibility.

Criterion	Inclusion	Exclusion
Scope of research	Empirical studies	Not empirical studies
Type of documents	Peer-reviewed articles	Not peer-reviewed articles
Written language	English	Not English
Research topic	Educators’ digital health	Not educators’ digital health
Full-text accessibility	Full text accessible	No full text

**Table 2:** Characteristics of the studies included in this scoping review of digital health among educators. The table presents first author, publication year, region, continent, sample size, and educational setting for each included study.

Citation	Year	Region	Continent	Sample Size	Educational Setting
Çelik Çoban & Büyükdereli Atadag [32]	2026	Turkey	Asia	325	Preschool
Khancolabi et al. [33]	2025	Iran	Asia	253	Primary school
Koştu & Arslan [34]	2026	Turkey	Asia	190 (79 teachers)	Higher education
Peralta et al. [35]	2026	Australia	Oceania	65	Primary and secondary school
Rangnow et al. [36]	2024	Germany	Europe	1600	Primary and secondary school
Yang & Shi [37]	2026	China	Asia	34	Primary and secondary school
Demir et al. [38]	2023	Turkey	Asia	223	Not specified (“Not specified” indicates that the relevant information was not clearly reported in the full text of the included article)
Feng et al. [39]	2026	China	Asia	3375	Kindergarten to secondary school
Huang et al. [40]	2022	Taiwan	Asia	485	Primary and secondary school
Rosário et al. [41]	2023	Portugal	Europe	853	Preschool
Kwon et al. [42]	2026	United States	North America	302 (223 teachers)	Preschool
Manning et al. [43]	2023	United Kingdom	Europe	8 (6 continued into the follow-up)	Secondary school
Hauck et al. [44]	2025	Germany	Europe	399	Primary and secondary school
Hirshberg et al. [45]	2022	United States	North America	662 (338 teachers)	Preschool to high school
Scanferla et al. [46]	2025	Brazil	South America	277	Primary school
Klatt et al. [47]	2025	Germany	Europe	81	Not specified
Shirouzu et al. [48]	2015	Japan	Asia	29	Kindergarten
Jalani et al. [49]	2026	Philippines	Asia	384	Not specified
Tachie-Donkor et al. [50]	2025	Ghana	Africa	20	Higher education
Grillo [51]	2021	United States	North America	82	Primary and secondary school
Kayabınar et al. [52]	2021	Turkey	Asia	40 (18 in the telerehabilitation training)	Primary and secondary school
Liu et al. [53]	2024	China	Asia	35	Primary school
Saintila et al. [54]	2021	Peru	South America	83	Higher education
Vidal-Conti & Galmes-Panades [55]	2025	Spain	Europe	61	Primary school
Manning et al. [56]	2020	United Kingdom	Europe	14	High school
Hsu et al. [57]	2019	Taiwan	Asia	534	Preschool
MacIsaac et al. [58]	2024	Scotland	Europe	12	Secondary school
Manning et al. [59]	2022	United Kingdom	Europe	14	High school
Yin et al. [60]	2023	China	Asia	Not specified	Primary school
Manning et al. [61]	2024	United Kingdom	Europe	8	High school
Musakuro & Gie [62]	2024	South Africa	Africa	348 (205 teachers)	Higher education

**Table 3:** Study designs and data collection methods of the studies included in this scoping review of digital health among educators.

Citation	Study Design	Data Collection Method(s)
Çelik Çoban & Büyükdereli Atadag [32]	Quantitative	Survey
Khancolabi et al. [33]	Quantitative	Survey
Koştu & Arslan [34]	Quantitative	Survey
Peralta et al. [35]	Quantitative	Survey
Rangnow et al. [36]	Quantitative	Survey
Yang & Shi [37]	Qualitative	Interview
Demir et al. [38]	Quantitative	Survey
Feng et al. [39]	Quantitative	Survey
Huang et al. [40]	Quantitative	Survey
Rosário et al. [41]	Quantitative	Survey
Kwon et al. [42]	Quantitative	Observation; Physiological evaluation; Survey
Manning et al. [43]	Qualitative	Interview; Survey
Hauck et al. [44]	Quantitative	App log data; Physiological evaluation; Survey
Hirshberg et al. [45]	Quantitative	Survey
Scanferla et al. [46]	Quantitative	Survey
Klatt et al. [47]	Quantitative	Interview; Physiological evaluation; Survey
Shirouzu et al. [48]	Quantitative	Physiological evaluation
Jalani et al. [49]	Quantitative	Survey
Tachie-Donkor et al. [50]	Qualitative	Interview
Grillo [51]	Quantitative	Physiological evaluation; Survey
Kayabınar et al. [52]	Quantitative	Survey
Liu et al. [53]	Quantitative	Clinician evaluation; Physiological Evaluation; Survey
Saintila et al. [54]	Quantitative	Survey
Vidal-Conti & Galmes-Panades [55]	Quantitative	Survey
Manning et al. [56]	Qualitative	Interview
Hsu et al. [57]	Quantitative	Survey
MacIsaac et al. [58]	Qualitative	Audio recording; Visual mind mapping
Manning et al. [59]	Qualitative	Empirical taxonomy-development study using a systematic review as one data source
Yin et al. [60]	Mixed-method design	Physiological evaluation; Survey
Manning et al. [61]	Qualitative	Interview; Survey
Musakuro & Gie [62]	Quantitative	Survey

**Table 4:** Findings of the Reviewed Articles.

Article	Findings
Çelik Çoban & Büyükdereli Atadag [32]	Preschool teachers demonstrated moderate digital health literacy, with protecting privacy representing the greatest challenge and adding content being the strongest skill. Higher digital health literacy was associated with greater infodemic awareness.
Khancolabi et al. [33]	Primary school teachers demonstrated digital health literacy below the standard level. Higher digital health literacy was significantly associated with greater health concerns, regular physical activity, general health knowledge, higher internet usage, and the use of social networks for health information.
Koştu & Arslan [34]	Higher education teachers demonstrated moderate levels of digital health literacy. Digital health literacy positively predicted healthy lifestyle behaviors, and it was a stronger predictor than AI literacy.
Peralta et al. [35]	Pre-service teachers generally demonstrated high digital health literacy, though scores were relatively lower regarding feeling safe and in control when using digital health services. Regional/institutional differences were observed regarding the usability and fit of digital services.
Rangnow et al. [36]	Primary and secondary school teachers experienced the greatest difficulties in protecting privacy and evaluating the reliability of digital health information. Digital health literacy subscales varied by gender and leadership roles.
Yang & Shi [37]	The “Digital Health Literacy Framework for PE Teachers” comprises three core dimensions: (1) Digital Health Awareness and Critical Literacy, (2) Digital Teaching Practice and Innovative Application Competence, and (3) Digital Security Ethics and Environmental Awareness Competence.
Demir et al. [38]	Digital health literacy differed by age and teaching experience, with the highest scores observed among teachers aged 30–39 years and those with 10–19 years of teaching experience. Higher digital health literacy was associated with greater cyberchondria severity.
Feng et al. [39]	Higher digital health literacy was associated with greater cognition of cancer warning symptoms and risk factors among basic education teachers, partially mediating the relationship between general self-efficacy and cancer cognition.
Huang et al. [40]	Digital health literacy differed by age, with younger teachers (aged 22–31) demonstrating higher functional digital health literacy than older teachers (aged 42–51). Higher functional digital health literacy was associated with better medication adherence.
Rosário et al. [41]	Digital health literacy, particularly information searching and determining relevance, was positively associated with early childhood teachers’ well-being. This relationship was partially mediated by teacher–child interaction.
Kwon et al. [42]	A multi-tiered intervention combining wearable devices, online modules, wellness coaching, and workplace environment enhancements significantly improved preschool teachers’ physical, psychological, and professional well-being.
Manning et al. [43]	High school teachers using consumer technologies (smartwatches, AI chatbots, mood/survey apps) experienced objective data as validating work demands, generating self-compassion, intrinsic permission to destress, and empathy for colleagues, leading to sustained, intentional self-care routines.
Hauck et al. [44]	Evaluating the app-based voice training mobile application for teacher vocal health in a randomized controlled trial showed high usability. The app significantly improved objective voice capability (DSI; Dysphonia Severity Index), and higher engagement was associated with lower DSI scores.
Hirshberg et al. [45]	A 4-week randomized controlled trial of the smartphone-based meditation/mindfulness training significantly reduced psychological distress (depression, anxiety) and improved psychological well-being, mindfulness, self-compassion, and work-related well-being among teachers.
Scanferla et al. [46]	The “Health and Voice” mHealth web application demonstrated high usability and high acceptability among primary school teachers. Its use significantly increased self-reported knowledge and self-awareness regarding vocal care.
Klatt et al. [47]	The fitness trackers successfully assessed teachers’ physiological stress (heart rate) across five intervals in a laboratory micro-teaching study, demonstrating significant heart rate increases prior to and during disruptive classroom situations followed by post-teaching recovery.
Shirouzu et al. [48]	Small wearable ECG and acceleration sensors were used to assess physiological stress by monitoring autonomic nervous system activity among kindergarten teachers. One teacher was found to have severe sleep apnea and autonomic nervous system activity levels comparable to those of patients with clinical depression or adjustment disorder.
Jalani et al. [49]	An AI-enabled workplace health kiosk deployed in a low-resource public school setting demonstrated high user acceptability and usability. Following 1–2 weeks of use, 70.9% of teachers consulted a healthcare provider and 66.7% made lifestyle changes.
Tachie-Donkor et al. [50]	University faculty members used AI-driven smart health devices for health monitoring, real-time feedback, and lifestyle motivation. While promoting health literacy and proactive habits, participants emphasized the need for clinical medical confirmation, data privacy safeguards, and managing continuous self-monitoring stress.
Grillo [51]	A global voice prevention program delivered via in-person or telepractice to pre-service teachers significantly improved acoustic voice parameters and reduced voice handicap and vocal fatigue compared to controls.
Kayabınar et al. [52]	Online teaching during COVID-19 significantly increased teachers’ musculoskeletal discomfort, disability, and depression/anxiety scores. Preventive telerehabilitation significantly reduced musculoskeletal discomfort, disability, and pain frequency.
Liu et al. [53]	Telepractice voice therapy delivered via smartphone videoconferencing to female primary school teachers with voice disorders achieved improvements in acoustic parameters, maximum phonation time, aerodynamic measures, and Voice Handicap Index scores equal to face-to-face therapy.
Saintila et al. [54]	A telehealth-based nutritional education program significantly increased nutritional knowledge and significantly decreased body weight and BMI among university teachers.
Vidal-Conti & Galmes-Panades [55]	An online school-based postural education intervention delivered via a digital platform significantly improved primary and secondary school teachers’ knowledge regarding back care, ergonomics, and low back pain prevention compared to baseline and controls.
Manning et al. [56]	14 midcareer high school teachers in England revealed that while 12 participants used consumer technologies to self-manage physical or psychological well-being, physical and cultural constraints in UK secondary schools (relentless schedules, lack of private space, and stigma surrounding “not coping”) restricted app use during the school day; leadership endorsement was identified as a key facilitator for technology adoption.
Hsu et al. [57]	Online learning communities (OLC) enhanced mental health literacy among preschool teachers, with teachers’ enthusiasm for engagement moderating the positive relationship between OLC participation and mental health literacy.
MacIsaac et al. [58]	Teachers engaged with complex “digital health assemblages”. Digital health mind mapping enabled teachers to visualize connections and served as a creative pedagogical tool to foster critical reflection on technology and health.
Manning et al. [59]	The study developed and populated a taxonomy mapping stress self-management strategies (educational, physiological, cognitive, social) against digital companion concepts (fostering reflection, guided self-management, peer support, entertainment) to support systematic technology choice for teachers.
Yin et al. [60]	The study constructed a health literacy index system for Chinese rural physical education teachers and showed that digital health empowerment had a direct and positive effect on teachers’ health literacy competencies and health education capabilities.
Manning et al. [61]	The study identified facilitators of teachers’ engagement with consumer technologies for stress management (passive data collection, brief interactions, reminders, data visualization, leadership support) and barriers (lack of instructions, connectivity issues, time pressures, workplace stigma, lack of privacy).
Musakuro & Gie [62]	Positive experiences with digital mental health services during COVID-19 were associated with perceived usefulness, user satisfaction, institutional commitment, service availability, and ethical considerations, while the absence of a legal framework and ethical/privacy concerns acted as primary barriers.

## Data Availability

No new data were created or analyzed in this study. Data sharing is not applicable to this article.

## Supplementary Materials

The following supporting information can be downloaded at: https://www.mdpi.com/article/10.3390/healthcare14193313/s1, Table S1: List of databases and search strings; Table S2: Preferred Reporting Items for Systematic reviews and Meta-Analyses extension for Scoping Reviews (PRISMA-ScR) Checklist; Table S3: PRISMA-S Checklist.

## Abbreviations

AI	Artificial intelligence
BMI	Body mass index
