# Supplementary material for: Digital Health Among Educators: A Scoping Review
Source: Healthcare (Basel). 2026 Oct 4;14(19):3313. doi: 10.3390/healthcare14193313 (PMC13665240; doi:10.3390/healthcare14193313)
Supplement: Supplementary file 1 [file healthcare-14-03313-s001.zip › Table S1.pdf]

Table S1. List of databases and search strings.

Database	Search date	Search string	Limits/filters
Scopus	8 June 2026	TITLE-ABS-KEY ((educator* OR teacher* OR instructor* OR professor* OR lecturer* OR tutor* OR "academic staff" OR "faculty member*" OR "teaching staff" OR "teacher educator*" OR "school staff" OR "education personnel" OR "academic personnel") AND ("digital health" OR ehealth* OR "e-health*" OR "e health" OR mhealth* OR "m-health*" OR "mobile health" OR telehealth* OR telemedicine* OR "digital health literacy" OR "ehealth literacy" OR "e-health literacy" OR "e health literacy" OR "health app*" OR "mobile health app*" OR ("wearable*" W/3 health) OR "fitness tracker*"))	No limits
Web of Science Core Collection	8 June 2026	TS=((educator* OR teacher* OR instructor* OR professor* OR lecturer* OR tutor* OR "academic staff" OR "faculty member*" OR "teaching staff" OR "teacher educator*" OR "school staff" OR "education personnel" OR "academic personnel") AND ("digital health" OR ehealth* OR "e-health*" OR "e health" OR mhealth* OR "m-health*" OR "mobile health" OR telehealth* OR telemedicine* OR "digital health literacy" OR "ehealth literacy" OR "e-health literacy" OR "e health literacy" OR "health app*" OR "mobile health app*" OR (wearable* NEAR/3 health) OR "fitness tracker*"))	No limits
PubMed	8 June 2026	((educator*[Title/Abstract] OR teacher*[Title/Abstract] OR instructor*[Title/Abstract] OR professor*[Title/Abstract] OR lecturer*[Title/Abstract] OR tutor*[Title/Abstract] OR "academic staff"[Title/Abstract]	No limits

		OR "faculty member"[Title/Abstract] OR "faculty members"[Title/Abstract] OR "teaching staff"[Title/Abstract] OR "teacher educator"[Title/Abstract] OR "teacher educators"[Title/Abstract] OR "school staff"[Title/Abstract] OR "education personnel"[Title/Abstract] OR "academic personnel"[Title/Abstract] OR "Faculty"[MeSH] OR "School Teachers"[MeSH]) AND ("digital health"[Title/Abstract] OR ehealth*[Title/Abstract] OR "e-health"[Title/Abstract] OR "e health"[Title/Abstract] OR mhealth*[Title/Abstract] OR "m-health"[Title/Abstract] OR "mobile health"[Title/Abstract] OR telehealth*[Title/Abstract] OR telemedicine*[Title/Abstract] OR "digital health literacy"[Title/Abstract] OR "ehealth literacy"[Title/Abstract] OR "e-health literacy"[Title/Abstract] OR "e health literacy"[Title/Abstract] OR "health app"[Title/Abstract] OR "health apps"[Title/Abstract] OR "mobile health app"[Title/Abstract] OR "mobile health apps"[Title/Abstract] OR wearable*[Title/Abstract] OR "fitness tracker"[Title/Abstract] OR "fitness trackers"[Title/Abstract] OR "Digital Health"[MeSH] OR "Telemedicine"[MeSH] OR "Wearable Electronic Devices"[MeSH] OR "Mobile Applications"[MeSH] OR "Health Literacy"[MeSH]))	
Embase	8 June 2026	(educator*:ti,ab,kw OR teacher*:ti,ab,kw OR instructor*:ti,ab,kw OR professor*:ti,ab,kw OR lecturer*:ti,ab,kw OR tutor*:ti,ab,kw OR 'academic staff':ti,ab,kw OR 'faculty	No limits

		member':ti,ab,kw OR 'faculty members':ti,ab,kw OR 'teaching staff':ti,ab,kw OR 'teacher educator':ti,ab,kw OR 'teacher educators':ti,ab,kw OR 'school staff':ti,ab,kw OR 'education personnel':ti,ab,kw OR 'academic personnel':ti,ab,kw OR 'teacher'/exp OR 'school teacher'/exp) AND ('digital health':ti,ab,kw OR ehealth*:ti,ab,kw OR 'e-health':ti,ab,kw OR 'e health':ti,ab,kw OR mhealth*:ti,ab,kw OR 'm-health':ti,ab,kw OR 'mobile health':ti,ab,kw OR telehealth*:ti,ab,kw OR telemedicine*:ti,ab,kw OR 'digital health literacy':ti,ab,kw OR 'ehealth literacy':ti,ab,kw OR 'e-health literacy':ti,ab,kw OR 'e health literacy':ti,ab,kw OR 'health app':ti,ab,kw OR 'health apps':ti,ab,kw OR 'mobile health app':ti,ab,kw OR 'mobile health apps':ti,ab,kw OR (('wearable' NEAR/3 health):ti,ab,kw) OR 'fitness tracker':ti,ab,kw OR 'fitness trackers':ti,ab,kw OR 'digital health'/exp OR 'telemedicine'/exp OR 'wearable electronic device'/exp OR 'mobile application'/exp OR 'health literacy'/exp)	
Academic Search Alumni Edition via EBSCOhost	8 June 2026	XB ((educator* OR teacher* OR instructor* OR professor* OR lecturer* OR tutor* OR "academic staff" OR "faculty member*" OR "teaching staff" OR "teacher educator*" OR "school staff" OR "education personnel" OR "academic personnel")) AND XB (("digital health" OR ehealth* OR "e-health*" OR "e health" OR mhealth* OR "m-health*" OR "mobile health" OR telehealth* OR telemedicine* OR "digital health literacy" OR "ehealth literacy" OR "e-health literacy" OR "e health literacy" OR "health	No limits

		app*" OR "mobile health app*" OR ("wearable*" N3 health) OR "fitness tracker*"))	
Academic Search Ultimate via EBSCOhost	8 June 2026	XB ((educator* OR teacher* OR instructor* OR professor* OR lecturer* OR tutor* OR "academic staff" OR "faculty member*" OR "teaching staff" OR "teacher educator*" OR "school staff" OR "education personnel" OR "academic personnel")) AND XB (("digital health" OR ehealth* OR "e-health*" OR "e health" OR mhealth* OR "m-health*" OR "mobile health" OR telehealth* OR telemedicine* OR "digital health literacy" OR "ehealth literacy" OR "e-health literacy" OR "e health literacy" OR "health app*" OR "mobile health app*" OR ("wearable*" N3 health) OR "fitness tracker*"))	No limits
APA PsycArticles via EBSCOhost	8 June 2026	XB ((educator* OR teacher* OR instructor* OR professor* OR lecturer* OR tutor* OR "academic staff" OR "faculty member*" OR "teaching staff" OR "teacher educator*" OR "school staff" OR "education personnel" OR "academic personnel")) AND XB (("digital health" OR ehealth* OR "e-health*" OR "e health" OR mhealth* OR "m-health*" OR "mobile health" OR telehealth* OR telemedicine* OR "digital health literacy" OR "ehealth literacy" OR "e-health literacy" OR "e health literacy" OR "health app*" OR "mobile health app*" OR ("wearable*" N3 health) OR "fitness tracker*"))	No limits
APA PsycBooks via EBSCOhost	8 June 2026	XB ((educator* OR teacher* OR instructor* OR professor* OR lecturer* OR tutor* OR "academic staff" OR "faculty member*" OR "teaching staff" OR "teacher educator*" OR "school staff" OR "education personnel" OR "academic personnel")) AND XB (("digital health" OR ehealth* OR "e-health*" OR "e health" OR	No limits

		mhealth* OR "m-health*" OR "mobile health" OR telehealth* OR telemedicine* OR "digital health literacy" OR "ehealth literacy" OR "e-health literacy" OR "e health literacy” OR "health app*" OR "mobile health app*" OR ("wearable*" N3 health) OR "fitness tracker*"))	
APA PsycInfo via EBSCOhost	8 June 2026	XB ((educator* OR teacher* OR instructor* OR professor* OR lecturer* OR tutor* OR "academic staff" OR "faculty member*" OR "teaching staff" OR "teacher educator*" OR "school staff" OR "education personnel" OR "academic personnel")) AND XB (("digital health" OR ehealth* OR "e-health*" OR "e health" OR mhealth* OR "m-health*" OR "mobile health" OR telehealth* OR telemedicine* OR "digital health literacy" OR "ehealth literacy" OR "e-health literacy" OR "e health literacy” OR "health app*" OR "mobile health app*" OR ("wearable*" N3 health) OR "fitness tracker*"))	No limits
Art & Architecture Source via EBSCOhost	8 June 2026	XB ((educator* OR teacher* OR instructor* OR professor* OR lecturer* OR tutor* OR "academic staff" OR "faculty member*" OR "teaching staff" OR "teacher educator*" OR "school staff" OR "education personnel" OR "academic personnel")) AND XB (("digital health" OR ehealth* OR "e-health*" OR "e health" OR mhealth* OR "m-health*" OR "mobile health" OR telehealth* OR telemedicine* OR "digital health literacy" OR "ehealth literacy" OR "e-health literacy" OR "e health literacy” OR "health app*" OR "mobile health app*" OR ("wearable*" N3 health) OR "fitness tracker*"))	No limits
Bibliography of Asian Studies via EBSCOhost	8 June 2026	XB ((educator* OR teacher* OR instructor* OR professor* OR lecturer* OR tutor* OR "academic staff" OR "faculty member*" OR	No limits

		"teaching staff" OR "teacher educator*" OR "school staff" OR "education personnel" OR "academic personnel")) AND XB (("digital health" OR ehealth* OR "e-health*" OR "e health" OR mhealth* OR "m-health*" OR "mobile health" OR telehealth* OR telemedicine* OR "digital health literacy" OR "ehealth literacy" OR "e-health literacy" OR "e health literacy" OR "health app*" OR "mobile health app*" OR ("wearable*" N3 health) OR "fitness tracker*"))	
British Education Index via EBSCOhost	8 June 2026	XB ((educator* OR teacher* OR instructor* OR professor* OR lecturer* OR tutor* OR "academic staff" OR "faculty member*" OR "teaching staff" OR "teacher educator*" OR "school staff" OR "education personnel" OR "academic personnel")) AND XB (("digital health" OR ehealth* OR "e-health*" OR "e health" OR mhealth* OR "m-health*" OR "mobile health" OR telehealth* OR telemedicine* OR "digital health literacy" OR "ehealth literacy" OR "e-health literacy" OR "e health literacy" OR "health app*" OR "mobile health app*" OR ("wearable*" N3 health) OR "fitness tracker*"))	No limits
British Education Index via EBSCOhost	8 June 2026	XB ((educator* OR teacher* OR instructor* OR professor* OR lecturer* OR tutor* OR "academic staff" OR "faculty member*" OR "teaching staff" OR "teacher educator*" OR "school staff" OR "education personnel" OR "academic personnel")) AND XB (("digital health" OR ehealth* OR "e-health*" OR "e health" OR mhealth* OR "m-health*" OR "mobile health" OR telehealth* OR telemedicine* OR "digital health literacy" OR "ehealth literacy" OR "e-health literacy" OR "e health literacy" OR "health app*" OR "mobile health app*"	No limits

		OR ("wearable*" N3 health) OR "fitness tracker*"))	
Business Source Elite via EBSCOhost	8 June 2026	XB ((educator* OR teacher* OR instructor* OR professor* OR lecturer* OR tutor* OR "academic staff" OR "faculty member*" OR "teaching staff" OR "teacher educator*" OR "school staff" OR "education personnel" OR "academic personnel")) AND XB (("digital health" OR ehealth* OR "e-health*" OR "e health" OR mhealth* OR "m-health*" OR "mobile health" OR telehealth* OR telemedicine* OR "digital health literacy" OR "ehealth literacy" OR "e-health literacy" OR "e health literacy” OR "health app*" OR "mobile health app*" OR ("wearable*" N3 health) OR "fitness tracker*"))	No limits
CINAHL via EBSCOhost	8 June 2026	XB ((educator* OR teacher* OR instructor* OR professor* OR lecturer* OR tutor* OR "academic staff" OR "faculty member*" OR "teaching staff" OR "teacher educator*" OR "school staff" OR "education personnel" OR "academic personnel")) AND XB (("digital health" OR ehealth* OR "e-health*" OR "e health" OR mhealth* OR "m-health*" OR "mobile health" OR telehealth* OR telemedicine* OR "digital health literacy" OR "ehealth literacy" OR "e-health literacy" OR "e health literacy” OR "health app*" OR "mobile health app*" OR ("wearable*" N3 health) OR "fitness tracker*"))	No limits
eBook Collection via EBSCOhost	8 June 2026	XB ((educator* OR teacher* OR instructor* OR professor* OR lecturer* OR tutor* OR "academic staff" OR "faculty member*" OR "teaching staff" OR "teacher educator*" OR "school staff" OR "education personnel" OR "academic personnel")) AND XB (("digital health" OR ehealth* OR "e-health*" OR "e health" OR mhealth* OR "m-health*" OR	No limits

		"mobile health" OR telehealth* OR telemedicine* OR "digital health literacy" OR "ehealth literacy" OR "e-health literacy" OR "e health literacy” OR "health app*" OR "mobile health app*" OR ("wearable*" N3 health) OR "fitness tracker*"))	
eBook Education Collection via EBSCOhost	8 June 2026	XB ((educator* OR teacher* OR instructor* OR professor* OR lecturer* OR tutor* OR "academic staff" OR "faculty member*" OR "teaching staff" OR "teacher educator*" OR "school staff" OR "education personnel" OR "academic personnel")) AND XB (("digital health" OR ehealth* OR "e-health*" OR "e health" OR mhealth* OR "m-health*" OR "mobile health" OR telehealth* OR telemedicine* OR "digital health literacy" OR "ehealth literacy" OR "e-health literacy" OR "e health literacy” OR "health app*" OR "mobile health app*" OR ("wearable*" N3 health) OR "fitness tracker*"))	No limits
eBook Open Access (OA) Collection via EBSCOhost	8 June 2026	XB ((educator* OR teacher* OR instructor* OR professor* OR lecturer* OR tutor* OR "academic staff" OR "faculty member*" OR "teaching staff" OR "teacher educator*" OR "school staff" OR "education personnel" OR "academic personnel")) AND XB (("digital health" OR ehealth* OR "e-health*" OR "e health" OR mhealth* OR "m-health*" OR "mobile health" OR telehealth* OR telemedicine* OR "digital health literacy" OR "ehealth literacy" OR "e-health literacy" OR "e health literacy” OR "health app*" OR "mobile health app*" OR ("wearable*" N3 health) OR "fitness tracker*"))	No limits
eBook University Press Collection via EBSCOhost	8 June 2026	XB ((educator* OR teacher* OR instructor* OR professor* OR lecturer* OR tutor* OR "academic staff" OR "faculty member*" OR "teaching staff" OR "teacher	No limits

		educator*" OR "school staff" OR "education personnel" OR "academic personnel")) AND XB (("digital health" OR ehealth* OR "e-health*" OR "e health" OR mhealth* OR "m-health*" OR "mobile health" OR telehealth* OR telemedicine* OR "digital health literacy" OR "ehealth literacy" OR "e-health literacy" OR "e health literacy” OR "health app*" OR "mobile health app*" OR ("wearable*" N3 health) OR "fitness tracker*"))	
Education Index Retrospective: 1929-1983 (H.W.Wilson) via EBSCOhost	8 June 2026	XB ((educator* OR teacher* OR instructor* OR professor* OR lecturer* OR tutor* OR "academic staff" OR "faculty member*" OR "teaching staff" OR "teacher educator*" OR "school staff" OR "education personnel" OR "academic personnel")) AND XB (("digital health" OR ehealth* OR "e-health*" OR "e health" OR mhealth* OR "m-health*" OR "mobile health" OR telehealth* OR telemedicine* OR "digital health literacy" OR "ehealth literacy" OR "e-health literacy" OR "e health literacy” OR "health app*" OR "mobile health app*" OR ("wearable*" N3 health) OR "fitness tracker*"))	No limits
Education Source Ultimate via EBSCOhost	8 June 2026	XB ((educator* OR teacher* OR instructor* OR professor* OR lecturer* OR tutor* OR "academic staff" OR "faculty member*" OR "teaching staff" OR "teacher educator*" OR "school staff" OR "education personnel" OR "academic personnel")) AND XB (("digital health" OR ehealth* OR "e-health*" OR "e health" OR mhealth* OR "m-health*" OR "mobile health" OR telehealth* OR telemedicine* OR "digital health literacy" OR "ehealth literacy" OR "e-health literacy" OR "e health literacy” OR "health app*" OR "mobile health app*"	No limits

		OR ("wearable*" N3 health) OR "fitness tracker*"))	
Environment Complete via EBSCOhost	8 June 2026	XB ((educator* OR teacher* OR instructor* OR professor* OR lecturer* OR tutor* OR "academic staff" OR "faculty member*" OR "teaching staff" OR "teacher educator*" OR "school staff" OR "education personnel" OR "academic personnel")) AND XB (("digital health" OR ehealth* OR "e-health*" OR "e health" OR mhealth* OR "m-health*" OR "mobile health" OR telehealth* OR telemedicine* OR "digital health literacy" OR "ehealth literacy" OR "e-health literacy" OR "e health literacy" OR "health app*" OR "mobile health app*" OR ("wearable*" N3 health) OR "fitness tracker*"))	No limits
ERIC via EBSCOhost	8 June 2026	XB ((educator* OR teacher* OR instructor* OR professor* OR lecturer* OR tutor* OR "academic staff" OR "faculty member*" OR "teaching staff" OR "teacher educator*" OR "school staff" OR "education personnel" OR "academic personnel")) AND XB (("digital health" OR ehealth* OR "e-health*" OR "e health" OR mhealth* OR "m-health*" OR "mobile health" OR telehealth* OR telemedicine* OR "digital health literacy" OR "ehealth literacy" OR "e-health literacy" OR "e health literacy" OR "health app*" OR "mobile health app*" OR ("wearable*" N3 health) OR "fitness tracker*"))	No limits
European Views of the Americas: 1493 to 1750 via EBSCOhost	8 June 2026	XB ((educator* OR teacher* OR instructor* OR professor* OR lecturer* OR tutor* OR "academic staff" OR "faculty member*" OR "teaching staff" OR "teacher educator*" OR "school staff" OR "education personnel" OR "academic personnel")) AND XB (("digital health" OR ehealth* OR "e-health*" OR "e health" OR mhealth* OR "m-health*" OR	No limits

		"mobile health" OR telehealth* OR telemedicine* OR "digital health literacy" OR "ehealth literacy" OR "e-health literacy" OR "e health literacy" OR "health app*" OR "mobile health app*" OR ("wearable*" N3 health) OR "fitness tracker*"))	
Funk & Wagnalls New World Encyclopedia via EBSCOhost	8 June 2026	XB ((educator* OR teacher* OR instructor* OR professor* OR lecturer* OR tutor* OR "academic staff" OR "faculty member*" OR "teaching staff" OR "teacher educator*" OR "school staff" OR "education personnel" OR "academic personnel")) AND XB (("digital health" OR ehealth* OR "e-health*" OR "e health" OR mhealth* OR "m-health*" OR "mobile health" OR telehealth* OR telemedicine* OR "digital health literacy" OR "ehealth literacy" OR "e-health literacy" OR "e health literacy" OR "health app*" OR "mobile health app*" OR ("wearable*" N3 health) OR "fitness tracker*"))	No limits
GreenFILE via EBSCOhost	8 June 2026	XB ((educator* OR teacher* OR instructor* OR professor* OR lecturer* OR tutor* OR "academic staff" OR "faculty member*" OR "teaching staff" OR "teacher educator*" OR "school staff" OR "education personnel" OR "academic personnel")) AND XB (("digital health" OR ehealth* OR "e-health*" OR "e health" OR mhealth* OR "m-health*" OR "mobile health" OR telehealth* OR telemedicine* OR "digital health literacy" OR "ehealth literacy" OR "e-health literacy" OR "e health literacy" OR "health app*" OR "mobile health app*" OR ("wearable*" N3 health) OR "fitness tracker*"))	No limits
Library, Information Science & Technology via EBSCOhost	8 June 2026	XB ((educator* OR teacher* OR instructor* OR professor* OR lecturer* OR tutor* OR "academic staff" OR "faculty member*" OR "teaching staff" OR "teacher	No limits

		educator*" OR "school staff" OR "education personnel" OR "academic personnel")) AND XB (("digital health" OR ehealth* OR "e-health*" OR "e health" OR mhealth* OR "m-health*" OR "mobile health" OR telehealth* OR telemedicine* OR "digital health literacy" OR "ehealth literacy" OR "e-health literacy" OR "e health literacy” OR "health app*" OR "mobile health app*" OR ("wearable*" N3 health) OR "fitness tracker*"))	
MAS Reference eBook Collection via EBSCOhost	8 June 2026	XB ((educator* OR teacher* OR instructor* OR professor* OR lecturer* OR tutor* OR "academic staff" OR "faculty member*" OR "teaching staff" OR "teacher educator*" OR "school staff" OR "education personnel" OR "academic personnel")) AND XB (("digital health" OR ehealth* OR "e-health*" OR "e health" OR mhealth* OR "m-health*" OR "mobile health" OR telehealth* OR telemedicine* OR "digital health literacy" OR "ehealth literacy" OR "e-health literacy" OR "e health literacy” OR "health app*" OR "mobile health app*" OR ("wearable*" N3 health) OR "fitness tracker*"))	No limits
MAS Ultra – School Edition via EBSCOhost	8 June 2026	XB ((educator* OR teacher* OR instructor* OR professor* OR lecturer* OR tutor* OR "academic staff" OR "faculty member*" OR "teaching staff" OR "teacher educator*" OR "school staff" OR "education personnel" OR "academic personnel")) AND XB (("digital health" OR ehealth* OR "e-health*" OR "e health" OR mhealth* OR "m-health*" OR "mobile health" OR telehealth* OR telemedicine* OR "digital health literacy" OR "ehealth literacy" OR "e-health literacy" OR "e health literacy” OR "health app*" OR "mobile health app*"	No limits

		OR ("wearable*" N3 health) OR "fitness tracker*"))	
MathSciNet via EBSCOhost	8 June 2026	XB ((educator* OR teacher* OR instructor* OR professor* OR lecturer* OR tutor* OR "academic staff" OR "faculty member*" OR "teaching staff" OR "teacher educator*" OR "school staff" OR "education personnel" OR "academic personnel")) AND XB (("digital health" OR ehealth* OR "e-health*" OR "e health" OR mhealth* OR "m-health*" OR "mobile health" OR telehealth* OR telemedicine* OR "digital health literacy" OR "ehealth literacy" OR "e-health literacy" OR "e health literacy" OR "health app*" OR "mobile health app*" OR ("wearable*" N3 health) OR "fitness tracker*"))	No limits
MEDLINE via EBSCOhost	8 June 2026	XB ((educator* OR teacher* OR instructor* OR professor* OR lecturer* OR tutor* OR "academic staff" OR "faculty member*" OR "teaching staff" OR "teacher educator*" OR "school staff" OR "education personnel" OR "academic personnel")) AND XB (("digital health" OR ehealth* OR "e-health*" OR "e health" OR mhealth* OR "m-health*" OR "mobile health" OR telehealth* OR telemedicine* OR "digital health literacy" OR "ehealth literacy" OR "e-health literacy" OR "e health literacy" OR "health app*" OR "mobile health app*" OR ("wearable*" N3 health) OR "fitness tracker*"))	No limits
Military & Government Collection via EBSCOhost	8 June 2026	XB ((educator* OR teacher* OR instructor* OR professor* OR lecturer* OR tutor* OR "academic staff" OR "faculty member*" OR "teaching staff" OR "teacher educator*" OR "school staff" OR "education personnel" OR "academic personnel")) AND XB (("digital health" OR ehealth* OR "e-health*" OR "e health" OR mhealth* OR "m-health*" OR	No limits

		"mobile health" OR telehealth* OR telemedicine* OR "digital health literacy" OR "ehealth literacy" OR "e-health literacy" OR "e health literacy” OR "health app*" OR "mobile health app*" OR ("wearable*" N3 health) OR "fitness tracker*"))	
Newspaper Source Plus via EBSCOhost	8 June 2026	XB ((educator* OR teacher* OR instructor* OR professor* OR lecturer* OR tutor* OR "academic staff" OR "faculty member*" OR "teaching staff" OR "teacher educator*" OR "school staff" OR "education personnel" OR "academic personnel")) AND XB (("digital health" OR ehealth* OR "e-health*" OR "e health" OR mhealth* OR "m-health*" OR "mobile health" OR telehealth* OR telemedicine* OR "digital health literacy" OR "ehealth literacy" OR "e-health literacy" OR "e health literacy” OR "health app*" OR "mobile health app*" OR ("wearable*" N3 health) OR "fitness tracker*"))	No limits
OpenDissertations via EBSCOhost	8 June 2026	XB ((educator* OR teacher* OR instructor* OR professor* OR lecturer* OR tutor* OR "academic staff" OR "faculty member*" OR "teaching staff" OR "teacher educator*" OR "school staff" OR "education personnel" OR "academic personnel")) AND XB (("digital health" OR ehealth* OR "e-health*" OR "e health" OR mhealth* OR "m-health*" OR "mobile health" OR telehealth* OR telemedicine* OR "digital health literacy" OR "ehealth literacy" OR "e-health literacy" OR "e health literacy” OR "health app*" OR "mobile health app*" OR ("wearable*" N3 health) OR "fitness tracker*"))	No limits
Primary Search via EBSCOhost	8 June 2026	XB ((educator* OR teacher* OR instructor* OR professor* OR lecturer* OR tutor* OR "academic staff" OR "faculty member*" OR "teaching staff" OR "teacher	No limits

		educator*" OR "school staff" OR "education personnel" OR "academic personnel")) AND XB (("digital health" OR ehealth* OR "e-health*" OR "e health" OR mhealth* OR "m-health*" OR "mobile health" OR telehealth* OR telemedicine* OR "digital health literacy" OR "ehealth literacy" OR "e-health literacy" OR "e health literacy" OR "health app*" OR "mobile health app*" OR ("wearable*" N3 health) OR "fitness tracker*"))	
Primary Search Reference eBook Collection via EBSCOhost	8 June 2026	XB ((educator* OR teacher* OR instructor* OR professor* OR lecturer* OR tutor* OR "academic staff" OR "faculty member*" OR "teaching staff" OR "teacher educator*" OR "school staff" OR "education personnel" OR "academic personnel")) AND XB (("digital health" OR ehealth* OR "e-health*" OR "e health" OR mhealth* OR "m-health*" OR "mobile health" OR telehealth* OR telemedicine* OR "digital health literacy" OR "ehealth literacy" OR "e-health literacy" OR "e health literacy" OR "health app*" OR "mobile health app*" OR ("wearable*" N3 health) OR "fitness tracker*"))	No limits
Regional Business News via EBSCOhost	8 June 2026	XB ((educator* OR teacher* OR instructor* OR professor* OR lecturer* OR tutor* OR "academic staff" OR "faculty member*" OR "teaching staff" OR "teacher educator*" OR "school staff" OR "education personnel" OR "academic personnel")) AND XB (("digital health" OR ehealth* OR "e-health*" OR "e health" OR mhealth* OR "m-health*" OR "mobile health" OR telehealth* OR telemedicine* OR "digital health literacy" OR "ehealth literacy" OR "e-health literacy" OR "e health literacy" OR "health app*" OR "mobile health app*"	No limits

		OR ("wearable*" N3 health) OR "fitness tracker*"))	
SPORTDiscus via EBSCOhost	8 June 2026	XB ((educator* OR teacher* OR instructor* OR professor* OR lecturer* OR tutor* OR "academic staff" OR "faculty member*" OR "teaching staff" OR "teacher educator*" OR "school staff" OR "education personnel" OR "academic personnel")) AND XB (("digital health" OR ehealth* OR "e-health*" OR "e health" OR mhealth* OR "m-health*" OR "mobile health" OR telehealth* OR telemedicine* OR "digital health literacy" OR "ehealth literacy" OR "e-health literacy" OR "e health literacy" OR "health app*" OR "mobile health app*" OR ("wearable*" N3 health) OR "fitness tracker*"))	No limits
Teacher Reference Center via EBSCOhost	8 June 2026	XB ((educator* OR teacher* OR instructor* OR professor* OR lecturer* OR tutor* OR "academic staff" OR "faculty member*" OR "teaching staff" OR "teacher educator*" OR "school staff" OR "education personnel" OR "academic personnel")) AND XB (("digital health" OR ehealth* OR "e-health*" OR "e health" OR mhealth* OR "m-health*" OR "mobile health" OR telehealth* OR telemedicine* OR "digital health literacy" OR "ehealth literacy" OR "e-health literacy" OR "e health literacy" OR "health app*" OR "mobile health app*" OR ("wearable*" N3 health) OR "fitness tracker*"))	No limits
The Serials Directory via EBSCOhost	8 June 2026	XB ((educator* OR teacher* OR instructor* OR professor* OR lecturer* OR tutor* OR "academic staff" OR "faculty member*" OR "teaching staff" OR "teacher educator*" OR "school staff" OR "education personnel" OR "academic personnel")) AND XB (("digital health" OR ehealth* OR "e-health*" OR "e health" OR mhealth* OR "m-health*" OR	

		"mobile health" OR telehealth* OR telemedicine* OR "digital health literacy" OR "ehealth literacy" OR "e-health literacy" OR "e health literacy" OR "health app*" OR "mobile health app*" OR ("wearable*" N3 health) OR "fitness tracker*"))	
